# Wellbeing in Workers during COVID-19 Pandemic: The Mediating Role of Self-Compassion in the Relationship between Personal Resources and Exhaustion

**DOI:** 10.3390/ijerph19031714

**Published:** 2022-02-02

**Authors:** Annalisa Grandi, Margherita Zito, Luisa Sist, Monica Martoni, Vincenzo Russo, Lara Colombo

**Affiliations:** 1 Department of Psychology, University of Turin, 10124 Turin, Italy; annalisa.grandi@unito.it (A.G.); lara.colombo@unito.it (L.C.); 2 Department of Business, Law, Economics and Consumer Behaviour “Carlo A. Ricciardi”, Università IULM, 20143 Milan, Italy; vincenzo.russo@iulm.it; 3 Department of Biomedical and Neuromotor Sciences, University of Bologna, 40138 Bologna, Italy; luisa.sist@unibo.it; 4 Department of Experimental, Diagnostic and Specialty Medicine, University of Bologna, 40138 Bologna, Italy; monica.martoni@unibo.it

**Keywords:** burnout, self-compassion, humor, optimism, occupational health, COVID-19

## Abstract

Italy was the second country to be affected by COVID-19 in early 2020, after China. The confrontation with the pandemic led to great changes in the world of work and, consequently, to the personal world of workers. In such a challenging situation, it is essential to be able to rely on resources that facilitate individual coping. The aim of this study was to understand the association between personal resources (optimism and humor) and exhaustion, and the role of self-compassion in this relationship. A structural equation model (SEM) was used to test the hypotheses on a heterogeneous sample of 422 Italian workers during the first lockdown in April–May 2020. The results revealed that optimism and humor were positively associated with self-compassion; optimism and humor also had a negative association with exhaustion; and self-compassion had a mediating role between the two personal resources and exhaustion. These results confirmed the importance of personal resources in maintaining workers’ wellbeing during a challenging period such as the pandemic. The present study also contributes to the body of knowledge on self-compassion, a relatively new construct that has been little studied in the organizational field.

## 1. Introduction

*Self-compassion is like a muscle.*

*The more we practice flexing it, especially when life doesn’t go exactly according to plan (a frequent scenario for most of us), the stronger and more resilient our compassion muscle becomes.*

[1]

Out of the blue, the year 2020 threw the world’s population into a situation of mixed confusion, fear, and uncertainty. The COVID-19 pandemic has led to a complete restructuring of the lives of millions of people in a very short period of time, forcing them to abandon established habits and lifestyles and face an unknown situation, at least in the memory of recent generations, and with the last historical precedent of similar magnitude dating back to the Spanish Flu [2]. The economic impact has been equally devastating: entire professional sectors (tourism, hotels, sports, entertainment, culture, etc.) have stopped working, as have many private businesses. All of this has inevitably led to major changes in the world of work as well—think of the massive and forced use of remote working for many categories of workers and the resulting impact on their personal lives—and consequently in the personal worlds of workers. COVID-19 has greatly affected the mental health of the world population, with strong psychological effects such as stress, anxiety, and depression [3,4,5]. In addition, the situation of obligatory quarantine resulted in negative psychological effects, post-traumatic stress disorder (PTSD) symptoms, anger, and confusion [6]. These negative effects on the mental health of the general population were also widely reported in epidemiological studies conducted in many countries affected by the epidemic [7,8,9,10].

Italy was the second country, immediately after China, to experience (largely unprepared) the devastating effects of COVID-19. According to the National Institute of Statistics, 45% of companies suspended their activities in the first months of the year due to the restrictions imposed by the national lockdown [11]. Studies conducted on national samples have confirmed poor sleep quality, high levels of anxiety, depression, and PTSD symptoms [5,12,13,14,15,16,17,18]. 

In a situation such as the one described above, it is essential to be able to draw on resources that can facilitate individual coping. These resources can be present in the work context, but can also be of a personal nature. The present study aims to understand how personal resources influenced workers’ experience of exhaustion during the first lockdown in Italy. Furthermore, the mediating role of personal resources such as self-compassion—that is a kind and mindful attitude towards oneself [19]—is explored.

## 2. Personal Resources at Work

According to the theoretical framework of job demand–resources theory [20], occupations are characterized by particular risks and protective factors, which can be divided into two main categories: demands and resources. Demands relate to features of work that require workers to exert effort (physical or psychological). Resources, on the other hand, are features of work that can help accomplish goals and stimulate personal development by buffering the impact of work demands. Resources can be of different nature, i.e., organizational, social, physical, or psychological. Personal resources are particularly helpful according to the conservation of resources theory (COR), and a lack of them can make individuals vulnerable to stressful events [21]. Consistent with COR theory, individuals who tend to maintain and protect their own personal resources are also more prone to developing new personal resources, following a gain cycle that helps them to effectively cope with the environment [21,22]. The literature has amply demonstrated how personal resources can contribute to the wellbeing of the worker. Their protective role against burnout has been studied in various occupational contexts, such as teachers [23,24], nurses [25,26,27], mental health workers [28,29], social workers [30], and academics [31]. In the organizational field, optimism is one of the most studied personal resources, due to its impact on the psychophysical state of individuals. Humor, on the other hand, which is of growing interest in the field of positive psychology, has been a less studied resource in the work context.

### 2.1. Optimism

Optimism can be broadly defined as a positive attitude toward life [32]. Optimistic people are indeed confident, not only in relation to a particular context, but more openly in relation to the challenges that life presents to them. Even in the face of difficult periods in life, when emotions such as anger, anxiety, and depression may arise, optimists are more likely to succeed in maintaining a good balance, because their confidence that things will go well can trigger a cycle of positive emotions [33]. The coping strategies of optimistic people seem to be associated, not only with problem-oriented strategies, but also with emotion-focused strategies. Optimists are able to see the best, even in critical situations, and can learn from the experience by focusing less on the negative aspects [33]. Furthermore, when the event they face is not changeable or controllable, they are able to accept it and adjust their view of the world to the new reality [33]. As shown in longitudinal studies, optimism is a trait [32]. However, it has also been shown to be related to the availability of resources in the individual’s developmental environment and how this can vary in certain situations [32]. One of the most widely used approaches to measuring optimism is to survey expectations; that is, to ask people directly whether or not their expectations are positive about life in general [32,34]. Interest in optimism was born in the field of health psychology, where it has been widely demonstrated that optimism has a positive effect on physical health [35] and also on longevity [36,37,38]. Other disciplines, such as occupational psychology, have also taken an interest in this construct and examined its role in the organizational context. Optimism has been found to be positively related to job satisfaction [39,40,41,42], work happiness [39], employee engagement [25,26,43], individual performance [42,43,44,45], team performance [46], and optimal experience at work, such as work flow [47]. Studies in organizational contexts have also shown that optimism plays a protective role and maintains the psychophysical wellbeing of workers. Indeed, it is negatively related to occupational stress [48] and burnout [25,26,49]. Recent studies have confirmed its protective role against exhaustion, even in the context of a pandemic [50].

### 2.2. Humor

Humor may be broadly seen as a lighthearted and non-serious outlook on ideas or life events [51], but actually has several psychological functions. When people experience positive emotions such as mirth—the emotion associated with humor—they can benefit both cognitively (e.g., more flexibility, more creative problem solving) and socially (e.g., more social responsibility, more prosocial behavior) [51]. Humor can also be useful when people want to communicate in a situation where it would be risky or uncomfortable to be too serious or direct [52]. In addition, humor reduces tension and is a good coping strategy during stress and difficult life events [53]. Studies have shown the protective role of humor on physical health (strengthening the immune system) and mental health (low depression, stress, and anxiety levels) [51,54,55,56,57]. Precisely because of its positive effects on individual health, humor has been studied in many areas of psychology, including more recently in occupational psychology. The protective role of humor in the workplace has been particularly studied in contexts where work involves the exposure to suffering and to situations with a strong emotional and traumatic impact. In studies conducted among emergency personnel [58], crime scene investigation [59], body handlers [60], and funeral workers [61], humor was demonstrated to be an effective coping strategy. A meta-analysis on the role of humor in the workplace has shown that it is positively associated with job performance, satisfaction, and group cohesion, and negatively associated with burnout, stress, and work withdrawal [62]. The inverse relationship between humor and exhaustion has also been highlighted in studies on employees [63], academics [64,65], school teachers [66], firefighters [67], doctors [68], and psychotherapists [69]. More recently, some studies have also shown the importance of humor as a coping strategy among workers in the pandemic context, highlighting its role in mitigating the perceived stress related to COVID-19 [70,71].

### 2.3. Self-Compassion

In the last decade, in particular, we have witnessed a growing scholarly production on self-compassion, especially with the aim of understanding its role in promoting and maintaining the wellbeing of individuals. Kristine Neff [19] began this line of studies and contributed to the definition of the construct in the scientific field. Self-compassion derives from the concept of compassion [72]; that is, being touched by the suffering of others, remaining in a state of awareness, allowing feelings of kindness toward them to arise, and desiring to mitigate their suffering. It also involves being non-judgmental towards others and being aware that fallibility is human. In this sense, self-compassion is the version of compassion directed toward oneself [19]. According to Neff [19], three dimensions help define the construct: self-kindness (being kind and benevolent to oneself), common humanity (viewing one’s experience as not isolated, but linked to human experience), and mindfulness (maintaining a balanced perspective and awareness of one’s experience, thoughts, and feelings). According to the author, self-compassion facilitates behaviors that are focused on one’s wellbeing [19]. 

To test this hypothesis, the author developed a scale to measure the level of self-compassion in individuals. It is an instrument consisting of 26 items divided into six subscales—self-kindness versus self-judgment, common humanity versus isolation, and mindfulness versus over-identification—that can also provide a total score of the person’s total self-compassion [73]. Although the author demonstrated the presence of six different factors at the factorial level [74], recent studies have shown that a two-factor structure of the scale, distinguishing between self-compassion and self-criticism (the latter also referred to as self-coldness or uncompassionate behavior) has a better fit [75,76,77,78,79,80,81,82]. Moreover, the ‘positive’ items seem to be better able to measure the construct of self-compassion [83,84], whereas the ‘negative’ items are more associated with psychopathological components [82,85,86]. Therefore, it was recommended to use only positive items to measure the protective nature of the construct [86,87]. According to Neff, these psychometric differences in the scale are partly due to translations of the original scale, and partly to possible cultural differences [74]. A new version of the scale, the Self-Compassion Scale–Short Form (SCS–SF), consisting of 12 items, has also been proposed [88], and which has a good internal consistency and high correlation with the original scale (with 26 items). A two-factor model was also developed for this scale, consisting of positive and negative items. Positive items were found to be a more accurate measure of the construct of self-compassion than negative items [86].

Compared to the results regarding individual wellbeing, as predicted by Neff [19,89], empirical research has shown that an attitude of self-compassion is a predictor of wellbeing [90,91,92] and is associated with various benefits: lower depression [73,93,94], lower anxiety [73,93,94], lower stress [94], lower perfectionism [73], and higher satisfaction with life [73,95,96]. Evidence related to gender showed that males have a slightly higher level of self-compassion than females [97]. Self-compassion has also been studied more recently in the field of occupational psychology, and it has been shown that those who have higher levels of self-compassion experience lower exhaustion [98,99,100,101,102,103] and higher job satisfaction [102,104]. In the organizational context, the mediating role of self-compassion in worker wellbeing has been poorly explored. For example, Reizer [105] showed that self-compassion is negatively correlated with dysfunctional attachment styles, turnover intentions, and exhaustion in the work context, and is instead positively correlated with job performance and organizational citizenship behaviors. In the study, the author also proved the role of self-compassion as a mediator in the relationship between the previous variables (attachment styles and outcome variables) [105]. Self-compassion is a very valuable resource, not only in daily life, but also in critical situations. Indeed, its importance in the pandemic context has been recognized in several international studies on samples of workers [106,107,108,109,110].

The first and the second hypotheses of our study concern the relationship between personal resources and self-compassion. More specifically:

**Hypothesis** **1** **(H1).** *Optimism is positively associated with self-compassion*.

**Hypothesis** **2** **(H2).** *Humor is positively associated with self-compassion*.

## 3. Exhaustion

Workplace features can have a major impact on workers’ mental health. Exhaustion is one of the main negative outcomes concerning the mental wellbeing of individuals at work. Considered the core component of the broader construct of burnout, exhaustion consists in the consumption of personal energy, due to an imbalance between job demands and the job and personal resources available to the individual. While exhaustion is mainly considered in its affective characteristics in the original conceptualization of Maslach’s burnout [111], Pine and colleagues [112] also considered the physical and mental aspects in demanding working conditions. In line with this last approach, the Oldenburg Burnout Instrument’s (OLBI) exhaustion dimension was formulated [113]. The OLBI operationalization of the exhaustion construct makes it possible to consider all energies of the worker, not only at the emotional, but also at the physical and the mental, level and can be extended to more professional sectors that also deal with physical and cognitive tasks [114]. According to its energy depleting feature, research has shown how exhaustion has a negative relationship with personal resources, such as optimism [25,26,49] and humor [63,64,65,66,67,68,69]. This negative association has also been confirmed referring to the pandemic work context [50].

The third and the fourth hypotheses of our study concern the relationship between personal resources and exhaustion. More specifically:

**Hypothesis** **3** **(H3).** *Optimism is negatively associated with exhaustion*.

**Hypothesis** **4** **(H4).** *Humor is negatively associated with exhaustion*.

Exhaustion has been studied in relation to another personal resource, i.e., self-compassion, and results also showed a negative association between the two constructs [98,99,100,101,102,103]. Self-compassion, in fact, seems to play an important role in facilitating individual coping, especially when challenging situations are faced at work.

The fifth, the sixth and the seventh hypotheses of our study concern the relationship between self-compassion and exhaustion and the role of self-compassion in the relationship between personal resources and exhaustion. More specifically:

**Hypothesis** **5** **(H5).** *Self-compassion is negatively associated with exhaustion*.

**Hypothesis** **6** **(H6).** *Self-compassion has a mediating role in the association between optimism and exhaustion*.

**Hypothesis** **7** **(H7).** *Self-compassion has a mediating role in the association between humor and exhaustion*.

In the light of this framework, the present study aimed to understand how personal resources (optimism and humor) influence self-compassion and exhaustion, and to investigate the mediating role of self-compassion between personal resources and exhaustion.

## 4. Materials and Methods

### 4.1. Study Design and Participants

The research project was approved by the Bioethics Committee of the University of Turin (protocol code no. 181450). Italian workers participated in the study between April and May 2020, a period in which government regulations in Italy placed the entire country in a state of national lockdown. An online questionnaire was created and a non-probability, purposive sample was employed, using the snowball sampling technique. Participants did not receive any compensation for completing the questionnaire.

In total, 422 questionnaires were collected, all valid. The respondents were all Italians. Most are women (70.6%), the mean age is 42.8 years (SD 10.7) with a range of 21–67 years (see Table 1). The cohabiting/married participants are 58.8% and 46.4% have children. At the time of the survey, 79.9% said they were living with someone. The majority of participants had at least a vocational diploma or high school education (34.9%) or a bachelor’s or master degree (42.9%). Regarding the professional sectors to which they belong, a large part of the sample came from Education and Research (19.2%) and Healthcare services (16.1%). Some participants (17.8%) reported practicing more than one profession. The average working seniority is 19 years (SD 11.2), with a range of >1–52 years. Most have a permanent employment contract (74.2%) and full-time working hours (77%). By contract, 67.3% of respondents can use remote working. Among the sample, 74.9% had never used remote working before COVID-19, 14.5% had used it but in an exceptional way, 7.1% used it regularly (e.g., 1/2 days every week or every month), and 2.8% work mainly with remote working. Regarding the work situation following the COVID-19 ordinances, 20.9% had been assigned new tasks/activities, 38.9% had to learn to use new technological tools, and 31.5%, given the peculiarity of their job, were forced to meet people (customers/patients) despite the restrictions.

### 4.2. Measures

The measurement scales used in the survey are validated tools that have demonstrated solid consistency and reliability in the literature. To assess the reliability of the scales, Chronbach’s α was calculated. All questions were in Italian.

**Personal resources**. Two personal resources were considered with a potential role of balancing exhaustion according to scientific evidence. 

*Optimism* was measured using 3 items of the Revised Life Orientation Test scale [34,115] on a 6-point response Likert scale (1 = disagreement, 6 = agreement); a sample item is: ‘In uncertain times, I usually expect the best’. 

*Humor* was measured using 6 items of the Humor Coping Scale [55,116] on a 4-point response Likert scale (1 = strongly disagree, 4 = strongly agree); a sample item is: ‘I have often found that my problems have been greatly reduced when I try to find something funny in them’.

**Outcome**. *Exhaustion* was measured using the 8-item scale of Oldenburg Burnout Inventory (OLBI) [114] on a 4-point response Likert scale (1 = strongly disagree, 4 = strongly agree); a sample item is: ‘During my work, I often feel emotionally drained’. 

**Mediator**. *Self-compassion* was measured using the 6 positive items of the Self-Compassion Scale–Short Form [88,117,118] on a 5-point response Likert scale (1 = never, 5 = very often); a sample item is: ‘I try to be understanding and patient towards those aspects of my personality I don’t like’.

### 4.3. Data Analysis

Correlations (Pearson’s r), alpha reliabilities (α) for each scale, and descriptive statistics were performed with SPSS 27. To test the mediating role of self-compassion between optimism and humor and exhaustion, a structural equations model (SEM) was estimated with MPLUS. These analyses provided a partial mediation model, and hypotheses were specified a-priori [119]. Goodness of fit of the model was evaluated using the chi-square value (χ2), the comparative fit index (CFI), the Tucker–Lewis index (TLI), the root mean square error of approximation (RMSEA), and the standardized root mean square residual (SRMR). The mediation in the model was assessed through indirect effects, which were also performed using a bootstrapping procedure. This technique in this model extracted 2000 new samples, to calculate the direct and indirect parameters of the model [120]. 

Moreover, the analyses performed in the SEM also used the parceling method to create latent variables. In fact, considering the high number of items, the latent variables of humor, self-compassion, and exhaustion were composed by two parcels (indicators composed by two or more items on average). The parceling method was employed to reduce type I errors in item correlations and to reduce the likelihood of a priori model misspecification [121,122]. All parcels in the present SEM obtained significant loadings (*p* < 0.001). 

As for the main model, in order to examine the potential effects of common method biases, two models were compared according the Harman’s single-factor procedure [123]. To do this, we first performed a confirmatory factor analysis considering the four latent variables, obtaining the following fit indices: χ^2^(21) = 36.142, *p* < 0.001, CFI = 0.99, TLI = 0.99, RMSEA = 0.04, SRMR = 0.02. Following this, this model was compared with a one-factor model with all items loading on one factor, which obtained the following fit indices: χ^2^(27) = 736.222, *p* < 0.000, CFI = 0.60, TLI = 0.47, RMSEA = 0.22, SRMR = 0.11. These results showed that the first model fit the data better than the one-factor model; thus, supporting the appropriateness of each item related to the hypothesized latent factor.

## 5. Results

From a psychometric standpoint, all the assessed variables in the study showed satisfactory Cronbach’s alphas, ranging between 0.78 and 0.82, all showing good reliability.

Correlations (Table 2) show that self-compassion was significantly and consistently correlated with all variables. Self-compassion showed positive correlations with optimism (r = 0.49) and humor (r = 0.39), and a negative correlation with exhaustion (r = −0.27). As for humor as a variable, it is interesting to underline the consistent data in correlations: in fact, humor was significantly and positively correlated with optimism (r = 0.37), and negatively correlated with exhaustion (r = −0.18).

The estimated structural equations model showed satisfactory fit indices, confirming the goodness of the model fit: χ^2^(21) = 36.142, *p* < 0.01, CFI = 0.99, TLI = 0.98, RMSEA = 0.04; C.I. 95% (01; 05); SRMR = 0.02. Moreover, the structural equations model showed parcels with significant loadings (*p* < 0.001).

**Figure 1:**
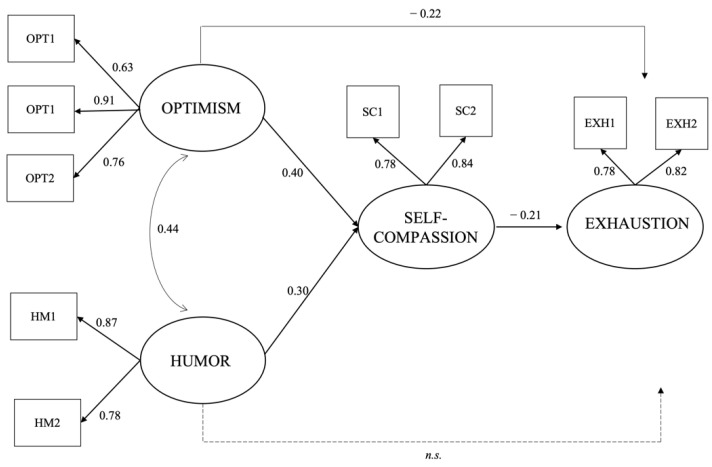
Results of the structural equations model. Note. (OPT = optimism; HM = humor; SC = self-compassion; EXH = exhaustion). OPT1 = item 1 of the latent variable optimism; OPT2 = item 2 of the latent variable optimism; OPT3 = item 3 of the latent variable optimism; HM1 = parcel 1 of the latent variable humor; HM2 = parcel 2 of the latent variable humor; SC1 = parcel 1 of the latent variable self-compassion; SC2 = parcel 2 of the latent variable self-compassion; EXH1 = parcel 1 of the latent variable exhaustion; EXH2 = parcel 2 of the latent variable exhaustion.

Deepening the model (Figure 1), optimism and humor were directly and positively associated with self-compassion (optimism: β = 0.40; humor: β = 0.30). While optimism was directly and negatively associated with exhaustion (β = −0.22), humor was not significantly associated with this variable. Moreover, the model showed a significant and negative association between self-compassion and exhaustion (β = −0.21).

In the present study, self-compassion was confirmed to have a mediating role. In fact, the model assessed negative, significant, and indirect associations between optimism and exhaustion through self-compassion (β = −0.10), and between humor and exhaustion through self-compassion (β = −0.05), showing a significant relationship between humor and exhaustion that in the direct effect was not significant, and confirming hypotheses H6 and H7. Table 3 shows these statistically significant indirect effects, obtained with the bootstrapping procedure (2000 replications).

## 6. Discussion

The present study analyzed the role of personal resources (optimism and humor) in relation to exhaustion during the pandemic COVID-19 and the role of self-compassion in this relationship. In particular, the results relate to the experiences of a heterogeneous sample of Italian workers—the second country, after China, to be affected after the advent of the pandemic—during the first quarter of 2020. 

Specifically, the aim of the present study was to test seven hypotheses, namely the positive association between personal resources (optimism and humor) and self-compassion (H1 and H2), the negative association of personal resources (optimism, humor, and self-compassion) with exhaustion (H3, H4 and H5); and the mediating role of self-compassion in the relationship between optimism and exhaustion (H6) and also in the relationship between humor and exhaustion (H7).

As for the first and the second hypotheses, our findings showed a positive and significant association between the personal resources included in the model, confirming H1 and H2. These findings are consistent with previous research that showed how optimism can predict self-compassion [124] and add new knowledge about the relationship between self-compassion and humor, not yet studied in the organizational field.

Regarding the third and the fourth hypotheses, both H3 and H4 were confirmed. In particular, the results showed that optimism has a significant negative association with exhaustion (H3), confirming its protective role in promoting health and wellbeing at work. This result is consistent with other international studies [25,26,49] and also with studies related to the pandemic context [50]. The analyses also revealed the negative association of humor with exhaustion (H4). This result supports the existing evidence in the literature on the functionality of this coping strategy against burnout [62,63,64,65,66,67,68,69], even in pandemic contexts [70,71].

The fifth hypothesis (H5), that is, self-compassion has a negative association with exhaustion, was also confirmed by the analyses. Self-compassion was, indeed, shown to be a factor able to counterbalance the negative experience of work exhaustion. This result is in line with findings from international research [98,99,100,101,102,103] and also with particular attention given to the COVID-19 work context [106,107,108,109,110].

The sixth and the seventh hypotheses (H6 and H7) were also confirmed by the analyses. Self-compassion, indeed, acts as a mediator in the relationships between optimism and exhaustion and between humor and exhaustion. While the negative association between self-compassion and burnout, particularly in the exhaustion dimension, has been widely reported in the literature [98,99,100,101,102,103], its mediating role in the context of personal resources has not yet been explored. For this reason, our findings are particularly useful, as they provide new insights into the construct of self-compassion in the organizational field. Furthermore, it is worth noting that, in our analyses, while a direct relationship between humor and exhaustion was detected, this relationship actually became significant when self-compassion was included in the model as a mediator. This result is particularly interesting, since it suggests that humor and self-compassion act in a sort of synergistic manner against the depleting effects of exhaustion. In particular, this finding supports the protective role of self-compassion against malaise at work and confirms the importance of pursuing and fostering this personal resource, as an effective coping strategy in work contexts. 

This study examined the relationship between personal resources and exhaustion during the pandemic COVID-19 and confirmed the protective role of personal resources in maintaining worker wellbeing. Therefore, how can these resources be supported and developed in the work context? Adopting interventions to support personal resources in the work context is a relatively new direction [125]. Developing an environment that fosters optimism, for example, by investing in training, can have an impact on organizational outcomes, as the literature has shown [41,126]. Commitment, satisfaction, and performance can increase, but most importantly, as we have seen in this study, psychological stress can be reduced, ensuring the psychophysical wellbeing of workers [41] and promoting organizational citizenship behavior [127]. As suggested by Riskind and colleagues, optimism training interventions should not only focus on reducing negative thoughts, but also on strengthening positive thinking [128]. 

Our study also highlighted the role of humor as an effective coping strategy in difficult contexts such as the pandemic. Again, proposing interventions aimed at developing humor among workers has already led to significant results, as a recent study shows [129].

Self-compassion has been shown to play an important role in the relationship between the two personal resources, optimism and humor, and exhaustion. Developing self-kindness means learning to think of oneself as worthy, even after disappointments or failures; it also means becoming more aware of one’s attitude toward self-judgment and the suffering it causes [91,130]. Realizing that we are not alone in times of suffering, but connected to others, is another quality of self-compassion, along with mindful attention to the present moment, without getting lost in emotions or thoughts, and, thus, being fully present [73]. When it is clear what immediate benefits this personal resource has for the individual in the private and social sphere, it becomes clear what results it could also have in the work context. Workers who are caring and understanding towards themselves may be less judgmental with themselves, but also with others, recognizing that everyone has flaws and makes mistakes. This awareness and the possibility of sharing one’s difficulties with one’s colleagues, would also facilitate interpersonal relationships, reinforcing the cohesiveness of the working group. In addition, in critical situations, bringing mindful attention to the present emotions and concerns would also help in maintaining a more balanced view and, therefore, to be more effective in problem solving. For these reasons, promoting interventions aimed at the development of self-compassion in work contexts can be a strategic investment, to support the wellbeing of workers and also of the organization. Several approaches have been proposed and some of these have proven their effectiveness, even in work contexts. One of the best known is Compassionate Mind Training (CMT), conceived by Paul Gilbert [130,131], which aims to develop compassion towards oneself through mindful attention training, soothing rhythm breathing, and compassionate imagery. Several studies have shown its effectiveness among workers [102,132,133,134] and also as an integration to education programs for future practitioners [135,136]. Another intervention strongly supported by scientific evidence is that of Mindfulness Based Stress Reduction (MBSR), developed by Joh Kabat-Zinn [137,138]. The program primarily focuses on raising awareness of the here-and-now, distancing oneself from the tendency of self-judging and intrusive and negative thoughts (rumination). A recent meta-analysis and systematic review reported many benefits for employees’ wellbeing associated with MBSR training; that is, a reduced level of exhaustion, occupational stress, depression, and anxiety [101,139]. 

Finally, it should be noted that, despite the contribution above discussed, the present study also has limitations. First, the sample was not very large and the non-probability sampling method can be affected by the subjectivity in the choice of participants. Second, the present study was a cross-sectional study, which does not allow highlighting certain relationships; to test causal relationships between variables, it would be important, in future, to use longitudinal models. Moreover, given the heterogeneity of the sample, in future research it might be interesting to analyze the differences and fluctuations of self-compassion between the different groups. Last, the use of only self-report measures may imply a risk of common method bias [140].

## 7. Conclusions

Our study helped to further understand the role that personal resources can have in counterbalancing negative outcomes in work contexts, especially in a very challenging period, such as a pandemic. These results also contribute to the recent line of research that wants to raise awareness in organizational contexts, to support and develop self-care practices among workers [141,142,143].

It is important to note that, while the short-term effects of the COVID-19 pandemic are already clearly visible, the long-term effects are not yet known. Based on previous situations that led to immediate and tragic changes in the lives of individuals, such as hurricanes, earthquakes, etc. [144,145], and also on studies highlighting the long-term negative effects of isolation situations [146], such as the that caused by the pandemic lockdown, it is possible to predict that managing post-COVID-19 mental health effects will be an issue of essential relevance in this year, but also in those to come. Therefore, looking forward, not only at the specific level of work contexts, it will be important that, in terms of public health, the government recognizes the strong impact that the COVID-19 pandemic has had on the entire national population, guaranteeing not only the protection of the physical, but also of the psychological, health of citizens. By making psychological support accessible to all citizens—considering, therefore, a greater integration of psychological services in primary healthcare—psychologists and psychotherapists could help citizens/patients, not only to elaborate the most painful and/or traumatic experiences related to the experience of the pandemic, but also in supporting their personal resources.

## Figures and Tables

**Table 1:** Descriptive statistics of the sample (N = 422).

	N	%
**Age** (M = 42.8 SD = 10.7)		
**Gender**		
Female	298	70.6
Male	124	29.4
**Relationship status**		
Single	143	33.9
Married/cohabiting	248	58.8
Separated/divorced	27	6.4
Widow/widower	4	0.9
**Education**		
Lower secondary school diploma	15	3.5
Vocational school diploma	13	3.1
High school diploma	134	31.8
Bachelor’s degree	30	7.1
Master’s degree	151	35.8
Post-graduate training	79	18.7
**Professional sectors**		
Agriculture and Handicraft	10	2.4
Business consulting	33	7.8
Culture, Sport and Tourism	33	7.8
Education and Research	81	19.2
Healthcare services	68	16.1
Industry	27	6.4
Mass media and Telecommunications	16	3.8
Public services and Administration	37	8.8
Social services	18	4.3
Trade/Commerce	37	8.8
Other	58	13.7
*Missing*	*4*	*0.9*
**Professional categories**		
Blue collar	15	3.6
Educator and Social Worker	10	2.4
Healthcare professional	46	10.9
Manager, Director	33	7.8
Scholar, Researcher, and Teacher	83	19.7
Self-employed professional	63	14.9
White collar	149	35.3
Other	19	4.5
*Missing*	*4*	*0.9*

**Table 2:** Means, Standard Deviations, and Correlations (Pearson’s r).

	M	SD	1	2	3	4
1. SELF-COMPASSION	3.28	0.72	(0.80)			
2. OPTIMISM	3.80	1.12	0.49 **	(0.78)		
3. HUMOR	2.75	0.85	0.39 **	0.37 **	(0.82)	
4. EXAUSTION	2.32	0.52	−0.27 **	−0.35 **	−0.18 **	(0.81)

Note. ** *p* < 0.01 level. Cronbach’s alphas are on the diagonal (between brackets).

**Table 3:** Indirect effects of the estimated SEM using bootstrapping (2000 replications).

Indirect Effects	Standardized Indirect Effects—Bootstrapping Procedure			
	Est.	s.e.	*p*	CI 95%
Optimism → Self-compassion → Exhaustion	−0.10	0.03	0.01	(−0.32, −0.04)
Humor → Self-compassion → Exhaustion	−0.07	0.03	0.00	(−0.10, −0.02)

## Data Availability

The data from the study are available on request. The data are not publicly available due to the Italian legislation on privacy.
